# Comparative metagenomic analysis of plasmid encoded functions in the human gut microbiome

**DOI:** 10.1186/1471-2164-11-46

**Published:** 2010-01-19

**Authors:** Brian V Jones, Funing Sun, Julian R Marchesi

**Affiliations:** 1 Centre for Biomedical and Health Sciences Research, School of Pharmacy and Biomolecular Sciences, University of Brighton, Lewes Road Brighton, UK; 2 Alimentary Pharmabiotic Centre and Dept of Microbiology, University College Cork, College Road, Cork, Ireland; 3 Cardiff School of Biosciences, Cardiff University, Park Place, Cardiff, UK

## Abstract

**Background:**

Little is known regarding the pool of mobile genetic elements associated with the human gut microbiome. In this study we employed the culture independent TRACA system to isolate novel plasmids from the human gut microbiota, and a comparative metagenomic analysis to investigate the distribution and relative abundance of functions encoded by these plasmids in the human gut microbiome.

**Results:**

Novel plasmids were acquired from the human gut microbiome, and homologous nucleotide sequences with high identity (>90%) to two plasmids (pTRACA10 and pTRACA22) were identified in the multiple human gut microbiomes analysed here. However, no homologous nucleotide sequences to these plasmids were identified in the murine gut or environmental metagenomes. Functions encoded by the plasmids pTRACA10 and pTRACA22 were found to be more prevalent in the human gut microbiome when compared to microbial communities from other environments. Among the most prevalent functions identified was a putative RelBE toxin-antitoxin (TA) addiction module, and subsequent analysis revealed that this was most closely related to putative TA modules from gut associated bacteria belonging to the *Firmicutes*. A broad phylogenetic distribution of RelE toxin genes was observed in gut associated bacterial species (*Firmicutes*, *Bacteroidetes*, *Actinobacteria *and *Proteobacteria*), but no RelE homologues were identified in gut associated archaeal species. We also provide indirect evidence for the horizontal transfer of these genes between bacterial species belonging to disparate phylogenetic divisions, namely Gram negative *Proteobacteria *and Gram positive species from the *Firmicutes *division.

**Conclusions:**

The application of a culture independent system to capture novel plasmids from the human gut mobile metagenome, coupled with subsequent comparative metagenomic analysis, highlighted the unexpected prevalence of plasmid encoded functions in the gut microbial ecosystem. In particular the increased relative abundance and broad phylogenetic distribution was identified for a putative RelBE toxin/antitoxin addiction module, a putative phosphohydrolase/phosphoesterase, and an ORF of unknown function. Our analysis also indicates that some plasmids or plasmid families are present in the gut microbiomes of geographically isolated human hosts with a broad global distribution (America, Japan and Europe), and are potentially unique to the human gut microbiome. Further investigation of the plasmid population associated with the human gut is likely to provide important insights into the development, functioning and evolution of the human gut microbiota.

## Background

The human gut harbours a complex microbial ecosystem which may encode ~100 times as many genes as the human genome and reaches a population density of 10^13^-10^14 ^cells in the distal colon [1-5]. This ecosystem is composed predominantly of bacteria belonging to the *Firmicutes *(mainly *Clostridia *and *Eubacteria*), and *Bacteroidetes *(mainly *Bacteroides*) [2,6], and it is estimated that the majority of species comprising this community (~80%) remain uncultured [3,6]. Metagenomic studies have begun to provide valuable insights into the metabolic activities undertaken by this community and the impact of this microbial ecosystem on host physiology and health [7-11].

Activities of the gut microbiota are the product of a long co-evolutionary relationship between host and microbe, which has resulted in a community that undertakes many functions beneficial to the human host [3,4]. The resident population of mobile genetic elements (MGE) associated with the human gut microbiota (the mobile metagenome) will also reflect the co-evolution of host and microbe in this community, with functions encoded by plasmids and other MGE shaped according to key environmental stresses, and host-microbe interactions important to life in the human gut [12,13].

Characterisation of cultivatable members of the human gut microbiota has highlighted the role of mobile genetic elements (MGE) in the spread of antibiotic resistance genes in this community [14-23]. In particular, the spread of erythromycin resistance genes among *Bacteriodes *sp. is well documented and attributed to mobilisation by conjugative transposons [14-16,18]. Evidence also exists for horizontal gene transfer (HGT) of tetracycline resistance genes in the gut microbial community [17-22]. Furthermore, the transfer of plasmids and transposons between diverse and disparate members of the mammalian gut microbiota has been demonstrated both *in vitro *and *in vivo *[17,23-25].

Genes involved in survival and persistence in the gastrointestinal tract, as well as activities that impact on host-microbe or microbe-microbe interactions, are also likely to be encoded by the gut mobile metagenome. As such elucidation of the functions encoded by the MGE associated with this community will be important in defining key aspects of this ecosystem. In addition, future attempts to manipulate this complex community for the enhancement of human health will require the genetic dissection of constituent species, for which plasmid vectors and other genetic tools are currently lacking. Exploitation of plasmids and other MGE populating the gut ecosystem will provide the raw genetic material for the development of such tools.

However, the large proportion of uncultivated species comprising the human gut microbiota has impeded study of this community, including the associated mobile metagenome. Overall little is known about the population of MGE that reside within this ecosystem, the genes and pathways they encode, or their contribution to ecosystem maintenance and function. Owing to the difficulty in accessing many members of this community, the application of culture-independent approaches to study MGE are required to further study this flexible gene pool. This has resulted in the development of metagenomic approaches aimed at investigating a range of MGE, including bacteriophage, plasmids, and integrons [13,26-28].

In this study we employed the culture independent transposon aided capture (TRACA) system to isolate novel plasmids from the human gut microbiota [13]. Subsequently a comparative metagenomic analysis was undertaken to investigate the distribution of these plasmids among individual gut metagenomes, as well as the relative abundance of plasmid encoded functions in the human gut microbiome.

## Results

### Plasmid annotation and prediction of encoded functions

**Figure 1:**
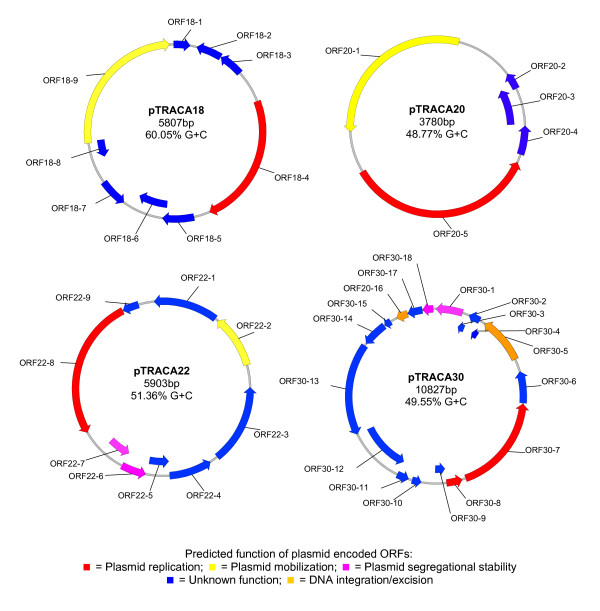
**Physical maps of complete nucleotide sequences for pTRACA18, pTRACA20, pTRACA22 and pTRACA30**. Maps of complete nucleotide sequences of plasmids pTRACA18, pTRACA20, pTRACA22, and pTRACA30 indicating locations of predicted open reading frames (ORFs). ORFs are colour coded according to predicted function as indicated in the associated key. Predicted products and more detail regarding putative functions of annotated ORFS are provided in **Table S1**.

Plasmids were acquired from the gut microbiome of a healthy volunteer using the TRACA system as previously described [13]. The complete nucleotide sequences of four plasmids designated pTRACA18, pTRACA20, pTRACA22 and pTRACA30 were obtained and annotated. These plasmids ranged from 3.78 kb to 10.827 kb in size with G+C contents of 48.77% to 60.5% (Fig 1).

Genes assigned putative functions in replication and mobilization were identified on all plasmids along with open reading frames (ORFs) of unknown function, many of which exhibited no significant homology to sequences present in the databases (Fig 1, Table S1 in additional file 1). This result indicates that a large number of uncharacterized activities, which may be relevant to ecosystem function, are encoded by plasmids in the gut community. For plasmids pTRACA18 and pTRACA20 the only functions encoded by these plasmids that could be assigned a putative function were those related to plasmid replication and mobilisation. However, further study is required to confirm the functional assignments of the genes encoded by these plasmids.

In the case of pTRACA22 and pTRACA30, ORFs predicted to encode systems involved in segregational stability and site specific integration or recombination were also identified (Fig 1, Table S1). Plasmid pTRACA30 was predicted to encode a putative ParA type ATPase (ORF30-1) with high identity to the ParA plasmid segregation protein from *Lawsonia intracellularis *Plasmid 2 (Accession number: NC_008013; Table S1), as well as a tyrosine site specific integrase (ORF30-5) belonging to the Int family of recombinases (Table S1). This integrase may mediate site specific recombination either within the same molecule of DNA or between distinct molecules of DNA [29]. As such, ORF30-5 may be involved in the resolution of plasmid dimers (which also enhances plasmid stability) [29], or mediate the site specific integration of pTRACA30 into the chromosome of the host bacterium. The presence of a putative excisionase on pTRACA30 (ORF30-8) strengthens this integration hypothesis.

Plasmid pTRACA22 was also predicted to encode genes involved in plasmid stabilization and a RelBE toxin/anti-toxin (TA) addiction module was identified (ORF22-6 and ORF22-7, Fig 1, Table S1). In addition, a putative entericidin toxin was also identified in this plasmid (ORF22-3) but no corresponding antitoxin was detected (Table S1). ORFs encoded by plasmid pTRACA22 exhibited a striking homology to sequences from the draft genome of *Blautia hydrogenotrophica *DSM 10507 (A member of the *Firmicutes *division isolated from human faeces). All pTRACA22 ORFS were found to be between 98-100% identical at the amino acid level to ORFs from *B. hydrogenotrophica *(Table 1). Homologous ORFs identified in *B*. *hydrogenotrophica *appeared to be contiguous based on gene locus identifiers (RUMHYD_1660, and RUMHYD_1665 to 1671; Table S1), however it is unclear at present if these correspond to a plasmid present in *B. hydrogenotrophica *as a finished genome sequence for this organism is currently unavailable.

**Table 1:** Identification of sequences homologous to plasmids in human gut metagenomes

Plasmid	Homologous metagenome sequence(s)^1 ^	Corresponding region of plasmid^2^	Identity	e-value
**pTRACA10**	Human 7			
	gb|AAQK01004081.1 (899)	4113 - 4577 (1)	91% (431/473)	6e-177
	Human F2-W			
	dbj|BAAY01023134.1 (1097)	3346 - 3966 (2)	82% (524/633)	6e-152
	Human In-D			
	dbj|BABD01003016.1 (1881)	3296 - 4096 (3)	82% (652/790)	0.0
**pTRACA18**	Human 8			
	gb|AAQL01009878.1 (2291)	47-209	90% (151/166)	3e-54
**pTRACA22**	Human 7			
	gb|AAQK01007350.1 (851) gb|AAQK01007351.1 (885) gb|AAQK01007353.1 (952) gb|AAQK01007354.1 (764)	96 - 942 (1) 1106 - 1941 (2) 3021 - 3967 (3) 4421 - 5183 (4)	83% (716/855) 88% (748/843) 91% (882/966) 89% (690/768)	0.0 0.0 0.0 0.0
	Human8			
	gb|AAQL01008280.1 (1257) gb|AAQL01008281.1 (3107) gb|AAQL01008279.1 (997)	846 -1941 (5) 2183 - 5503 (6) 5547 - 5744 (7)	89% (984/1104) 89% (2436/2733) 92% (185/201)	0.0 0.0 5e-72
	Human In-R			
	dbj|BABG01020620.1 (940) dbj|BABG01024736.1 (979) dbj|BABG01024737.1 (921) dbj|BABG01020619.1 (910)	162 - 1102 (8) 3377 - 4334 (9) 4293 - 5209 (9) 5503 - 5903 (10)	94% (890/946) 95% (915/960) 94% (872/922) 91% (376/411)	0.0 0.0 0.0 3e-154
	Human F2-W			
	dbj|BAAY01022991.1 (1098) dbj|BAAY01017891.1 (946) dbj|BAAY01006494.1 (1365) dbj|BAAY01019825.1 (1034) dbj|BAAY01002412.1 (1906)	1 - 602 (11) 479 - 945 (12)* 1390 - 1940 (13) 2813 - 3123 (14) 3865 - 5751 (15)	95% (578/603) 83% (795/950) 92% (512/552) 86% (272/314) 93% (1780/1897)	0.0 0.0 0.0 7e-91 0.0
	Human F2-V			
	dbj|BAAX01003632.1 (1386)	2718 - 4071 (16)	96% (1318/1362)	0.0
	Human F2-Y			
	dbj|BABA01021839.1 (940)	4042 - 4710 (17)	80% (550/682)	1e-132
	Human In-E			
	dbj|BABE01009723.1 (975)	3858 - 4653 (18)	83% (678/810)	0.0
	Human In-D			
	dbj|BABD01025538.1 (985)	3049 - 3966 (19)	94% (871/921)	0.0
	Human In-M			
	dbj|BABF01004953.1 (1116)	1310 - 1411 (20)	89% (95/106)	2e-27

**Figure 2:**
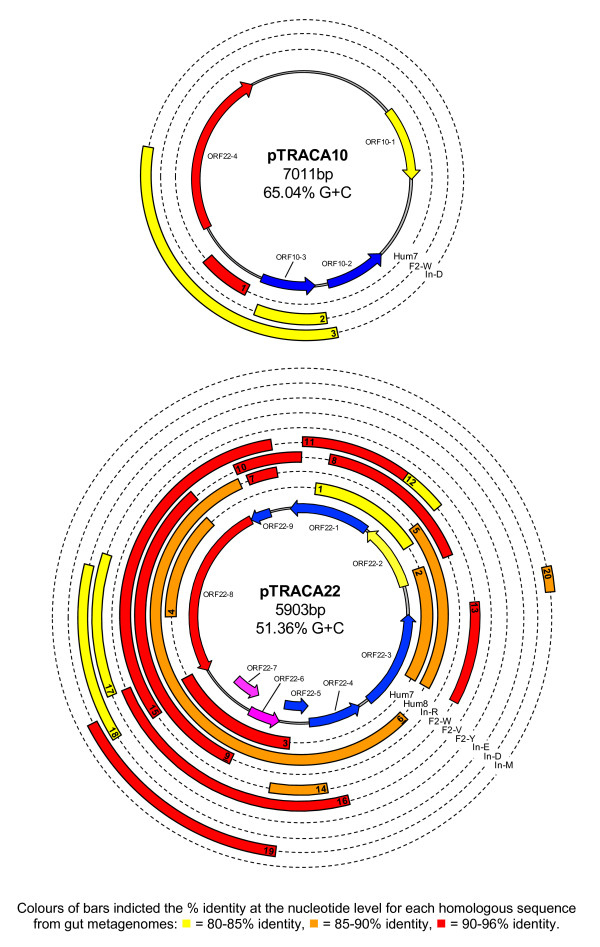
**Identification of sequences homologous to pTRACA10, pTRACA22 and encoded ORFS in human gut metagenomes**. The complete nucleotide sequences of plasmids was used to search 15 human gut metagenomes, the combined gut metagenome of lean and obese mice, Sargasso sea, and soil metagenomes [9-11,30,31]. The central ring shows the physical plasmid map with encoded ORFs, as in **Figure 1 **and **Table S1**. Concentric rings represent the nine human gut metagenomes in which homologous sequences were identified, and bars indicate regions of homology between sequences retrieved from human gut metagenomes and corresponding regions of the pTRACA10 or pTRACA22 plasmids. Colours of bars indicate the % identity at the nucleotide level for each metagenomic sequence as detailed in the associated key. Only sequences >100 bp in length are shown, and numerals within bars correspond to detailed information on the relevant metagenomic sequences provided in **Table 1**. Metagenomes are as follows: Hum7, Hum8 - American metagenomes [9]; In-R, F2-W, F2-V, F2-Y, In-E, In-D, In-M - Japanese metagenomes [10].

**Figure 3:**
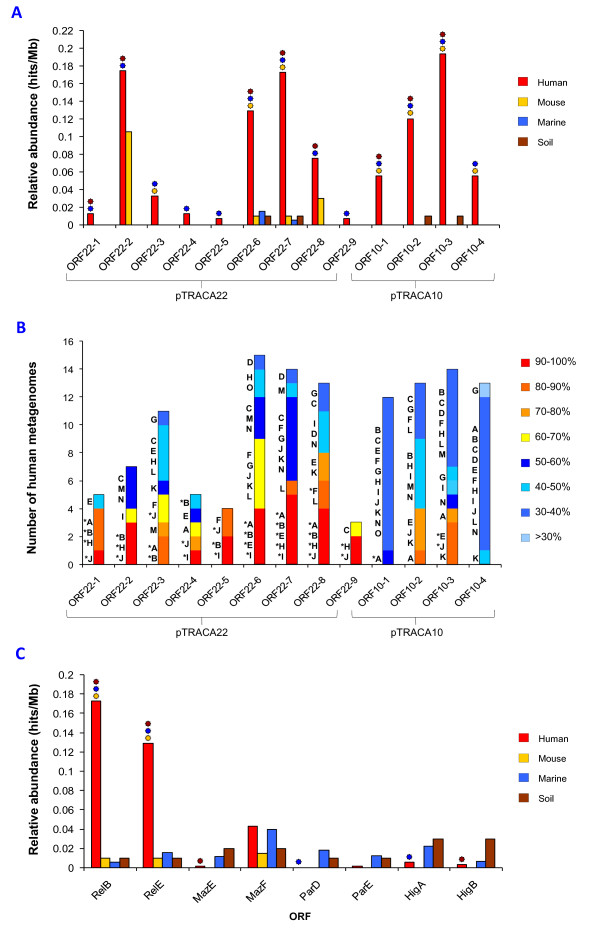
**Distribution and relative abundance of plasmid encoded functions in the human gut microbiome**. **A) **Relative abundance of pTRACA22 ORFs, expressed as hits/Mb, in the combined human gut metagenomes of 15 individuals [9,10] compared to the combined murine gut metagenome [11], Sargasso sea [30], and soil metagenomes [31]. Symbols above bars indicate significant differences between combined human metagenomes and each non-human metagenome (P = 0.01 or less), and colours correspond to each non-human metagenome: Orange = Significant difference between human and murine metagenomes; Blue = Significant difference between human and marine metagenomes; Brown = significant difference between human and soil metagenomes. **B) **Distribution of amino acid sequences homologous to ORFs encoded by pTRACA10 and pTRACA22 in 15 individual human gut metagenomes. Bars indicate the number of human metagenomes in which amino acid sequences homologous to each plasmid encoded ORF were detected. Colours within bars indicate the overall % identity of the top hits (based on bit score) in each metagenome to the corresponding plasmid ORF. Letters adjacent to bars indicate human metagenomes in which sequences homologous to plasmid ORFs were identified: **A**) Human7, **B**) Human8 [9]; **C) **InA, **D**) InB, **E**) InD, **F**) InE, **G**) InM, **H**) InR, **I**) F2-V, **J**) F2-W, **K) **F2-X, **L) **F2-Y, **M) **F1-S, **N) **F1-T, **O**) F1-U [10]. * indicates metagenomic sequences that correspond to those represented in **Figure 2 **and **Table 1**. **C) **Relative abundance (as hits/Mb) of the pTRACA22 RelBE TA module in human gut, murine gut and environmental metagenomes, compared to relative abundance of MazEF, ParDE and HigBA TA modules. The observed differences between human and non-human metagenomes were explored using the χ2 distribution. Symbols above bars indicate approximate significance of differences between combined human metagenomes and each non-human metagenomes (P = 0.01 or less), as in Fig 1a.

Human gut [9,10], murine gut [11], marine and soil [30,31] metagenomes were searched for sequences homologous to plasmid recovered from the human gut using Blastn. No significant homology was found for any plasmid in the non-human metagenomes. For plasmids pTRACA10, 18 and 22 homologous sequences were identified in human gut metagenomes. **1**) Homologous sequences identified in each human metagenome to plasmids. Accession numbers for individual metagenomic sequences are provided and figures in parentheses indicate the total length of each metagenomic sequence. **2**) Provides coordinates for the corresponding region of homology on relevant plasmids. Figures in parentheses indicate the corresponding fragment mapped onto plasmid sequences in Figure 2. *For region 12 (479-945 on plasmid pTRACA22) only the region from 602-945 bp on plasmid pTRACA22 was represented in Figure 3 due to overlap with region 11 (bp 1-602 on pTRACA22).

### Comparative metagenomic analysis of plasmids and encoded functions

The presence of these plasmids, and the relative abundance of their encoded functions in the human gut microbiome was investigated using metagenomic data sets derived from fifteen human gut microbiomes, the Sargasso Sea, soil, and the combined gut metagenomes of lean and obese mice [9-11,30,31]. Initially metagenomes were searched for nucleotide sequences homologous to newly characterised plasmids (pTRACA18, pTRACA20, pTRACA22, and pTRACA30), as well as two previously characterised plasmids (pTRACA10, and pTRACA17, [GenBank: AM263036, AM263037]) isolated from the same individual also using the TRACA system [13].

No significant hits were found for pTRACA17, pTRACA20, or pTRACA30 in any of the metagenomes examined, and only limited homology to pTRACA18 was observed in one human gut metagenome (Table 1). In contrast, for plasmids pTRACA22 and pTRACA10 nucleotide sequences with high identity to regions of these plasmids were detected in multiple human gut metagenomes which included both American and Japanese individuals (Fig 2, Table 1). Searches of marine, terrestrial, and murine gut metagenomes failed to identify any nucleotide sequences with significant homology to any of the plasmids analysed (Table 1).

Nucleotide sequences with high identity to pTRACA22 were detected in 9 of the 15 human gut metagenomes examined. In total 81.9% of the pTRACA22 nucleotide sequence was represented at 90% identity or greater in the collective human gut metagenomes examined, while 95.9% was represented at 80% identity or greater (Fig 2, Table 1). Nucleotide sequences with high identity to pTRACA10 were identified in 3 of the 15 human gut metagenomes searched, and these sequences all related to a distinct region of the pTRACA10 plasmid. This region encodes a putative phosphohydrolase/phophoesterase belonging to COG4186 (ORF10-3, Fig 2, Table 1), with high identity to an ORF from the draft genome sequence of *Collinsella aerofaciens *ATCC25986 (COLAER_02400, 85% identity, 5e^-108^).

Due to the detection of nucleotide sequences homologous to pTRACA10 and pTRACA22 among multiple human gut microbiomes, the relative abundance of functions encoded by these plasmids in the human gut microbiome was assessed. This analysis revealed that functions encoded by pTRACA10 and pTRACA22 are more common in the human gut microbiome when compared to the non-human metagenomic datasets analysed. Several of these ORFs were found to be significantly more prevalent in the combined human gut metagenomes when compared to murine, marine and soil metagenomes (Fig 3a-b). Of these the most highly enriched functions were the pTRACA10 phosphoesterase/phosphohydrolase (ORF10-3) along with ORF10-2 (unknown function), and the pTRACA22 RelBE toxin-antitoxin addiction module (ORF22-6 and ORF22-7; Fig 3a).

Variation between individuals was explored using searches of the 15 individual human gut metagenomes to identify amino acid sequences homologous to plasmid encoded ORFs. This revealed that ORF10-3, ORF22-6, and ORF22-7 also appeared to be widely distributed among the individual human gut metagenomes examined (Fig 3b). While the increased abundance of proteins involved in functions such as DNA replication are expected and in line with findings from other metagenomic studies [10], the significance of the increased prevalence observed for other plasmid encoded functions in this ecosystem is unclear.

In particular the greater incidence of RelBE addiction modules in the human gut microbiota was unexpected. TA modules have previously been demonstrated to be highly abundant in free living bacteria and archaea from a wide range of habitats, and absent in obligate intracellular prokaryotes [32]. As such the high relative abundance of the identified RelBE TA modules in the human gut microbiota compared to other metagenomic datasets is surprising, and may indicate the development of a gut associated RelBE sub type, rather than an absence of RelBE genes in microbial populations from other environments.

Alternatively, TA modules such as MazEF, ParDE, and HigBA may be more prevalent in natural environments with RelBE modules present at a lower abundance. To test this hypothesis the relative abundance in human gut, marine, terrestrial and murine metagenomes of TA modules from other families was assessed and compared to the pTRACA22 RelBE module (Fig 3c). No other TA modules were found to be as prevalent in the human gut microbiome as homologues of the pTRACA22 RelBE. The relative abundance of each module in non-human metagenomes were similar but generally lower in human gut metagenomes (Fig 3c). However, the MazF toxin component of MazEF modules appeared to be more prevalent in human gut metagenomes than the corresponding antitoxin or other TA genes, and this was also the case in the marine and murine metagenomes (Fig 3c).

### Phylogenetic analysis of pTRACA22 and metagenomic RelE sequences

To further explore the distribution of RelBE TA modules in the gut metagenome and the relationship between the pTRACA22 RelBE module, homologous TA modules from combined human gut metagenomes, and those encoded on plasmids or bacterial chromosomes, a phylogenetic analysis using RelE toxin components was undertaken.

Human gut metagenomic nucleotide sequences were retrieved and both *relE *and *relB *ORFs were identified (Table S2 in additional file 2). The majority of metagenomic sequences were found to encode complete *relBE *addiction modules exhibiting characteristic genetic architecture, in which *relB *genes are encoded upstream of and overlapping the start codon of the *relE *gene [32]. In the majority of sequences where a cognate *relB *was not detected this could be attributed to truncation of the sequence, or the presence of orphan toxin genes (Table S1). However several sequences were identified in which a suitable *relB *candidate ORF was detected up stream of the identified *relE *gene, but lacked RelB conserved domains or significant homology to RelB proteins (Table S2).

**Figure 4:**
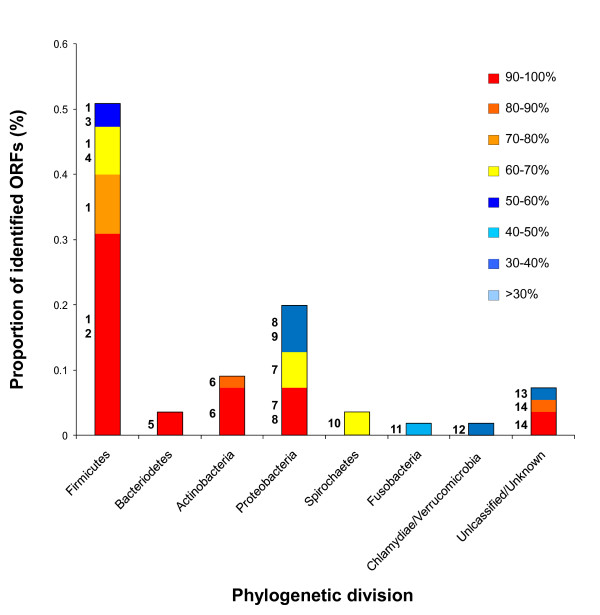
**Distribution of RelE genes encoded by sequences from human gut metagenomes**. RelE sequences homologous to pTRACA22 ORF22-6 identified in human gut metagenomes during comparative metagenomic analysis (Figure 3) were affiliated with a phylogenetic division based on genus represented in top hits (based on bit score) from BlastP and tBlastn searches of public databases encompassed by the nr dataset. Bars represent the proportion of RelE sequences identified which were affiliated with each phylogenetic division, and colours within bars indicate the % identity of metagenomic sequences to homologous sequences in bacterial genomes or plasmids. Numerals adjacent to bars indicate the genus in which homologous RelE were identified at each corresponding level of % identity: **1**) *Clostridium ***2**) *Faecalibacter ***3**) *Desulfitobacterium*, **4**) *Blautia*, **5**) *Parabacteroides*, **6**) *Bifidobacteria*, **7**) *Escherichia *(pARS3), **8**) *Yersinia*, **9**) *Photorhabdus*, **10**)*Treponema*, **11**) *Fusobacteria*, **12**) *Parachlamydia*, **13**) "*Candidatus *Cloacamonas", (Candidate division WWE1) **14**) Uncultured bacterium, fosmid clone 3 originating from organically reared pig gut and encoding tetracycline resistance [43].

The resulting set of 55 full length RelE homologues were initially affiliated with bacterial phylogenetic divisions based on the species identified in BlastP or tBlastn searches of the nr dataset (see materials and methods for details). The majority of RelE homologues identified were found to exhibit highest identity to sequences from bacteria belonging to the *Firmicutes*, (in particular the genus *Clostridium*) and *Proteobacteria *(Fig 4). *Firmicutes *have been estimated to represent a significant proportion of the gut microbiota and co-dominate this ecosystem with members of the *Bacteroidetes *division [2,6,33]. As such the large proportion of human gut metagenome derived RelE affiliated with this division is not unexpected, however, in comparison few sequences were affiliated with the *Bacteroidetes *division (Fig 4).

In contrast, *Proteobacteria *account for a relatively minor proportion of the adult gut microbiota [2,6,33], but may co-dominate with *Actinobacteria *in the infant gut [10]. Interestingly the majority of gut metagenomic RelE types assigned to this group exhibited high identity to the *Escherichia coli *pARS3 plasmid (Fig 4). The pARS3 plasmid was originally identified in a clinical isolate of *E. coli *designated ARS3, which was obtained from the urine of an inpatient in a Japanese general hospital [34]. Subsequent characterisation revealed pARS3 to be a large (~115 kb) conjugative plasmid encoding a novel 16S methyltransferase, that functions as a broad spectrum amino glycoside resistance determinant [34].

Overall, 33 distinct full length putative RelE "types" (designated type#1 to type#33) were retrieved from combined human gut metagenomes (Table S2), and aligned with the pTRACA22 RelE, and homologous plasmid and chromosomally encoded sequences retrieved using standard BlastP and tBlastn searches of the nr dataset (see materials and methods for details). Notably, searches of the public databases encompassed by the nr dataset did not identify any homologous sequences originating in archaea, and specific searches of the genome sequences of the gut associated archaeal species *Methoanobrevibacter smithii *and *Methanosphaera stadmanae*, also failed to identify any homologues.

**Figure 5:**
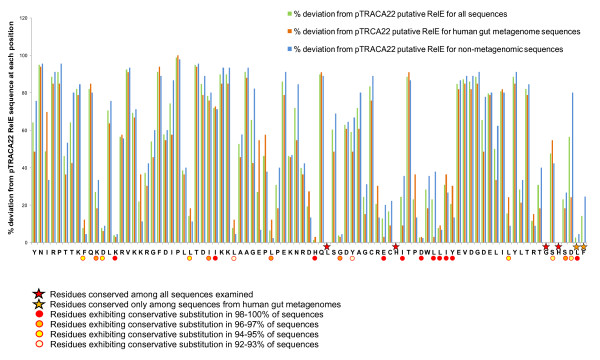
**Identification of conserved regions of RelE**. Plot showing the percentage divergence from the pTRACA22 RelE sequence of homologous RelE sequences. Bars represent the percentage divergence in identical amino acids at each position of the pTRACA22 RelE sequence, based on alignments of RelE sequences from human gut metagenomes, plasmids and bacterial chromosomes. The pTRACA22 sequence is presented with the N terminus at the origin of the plot, and the N terminal M start codon omitted. Symbols indicate conserved residues (stars) and those at which between 90 and 100% of represented sequences exhibit a conserved substitution (circles), as indicated by the associated key.

Alignments of RelE amino acid sequences revealed conserved residues, and highlighted regions of similarity as well as regions predominantly conserved among metagenomic sequences (Fig 5). Overall metagenomic sequences generally showed less divergence from the pTRACA22 RelE than other sequences included in the alignment, and the C terminus L and F residues appeared completely conserved among these sequences (Fig 5).

**Figure 6:**
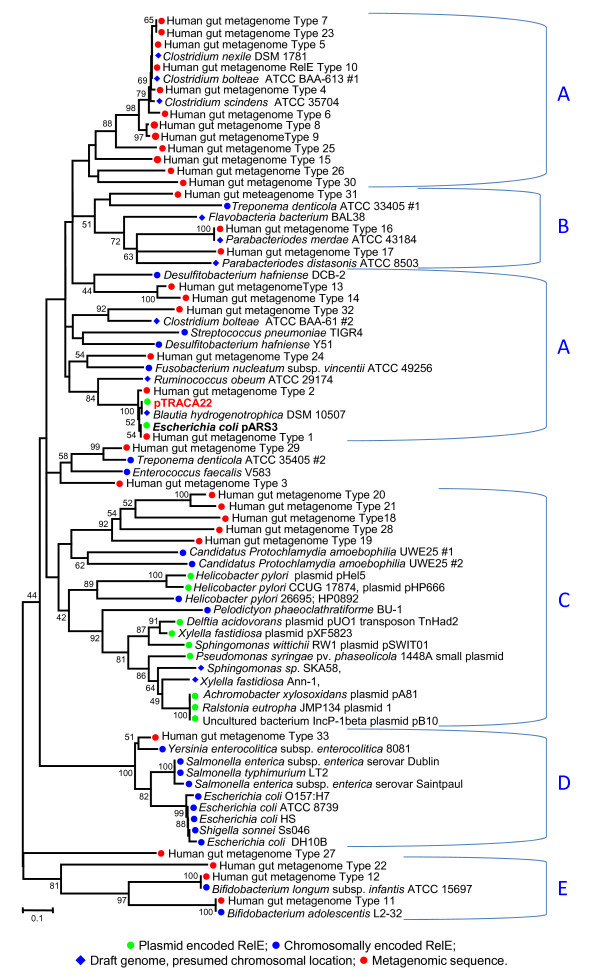
**Phylogenetic distribution of RelE toxin sequences**. Amino acid sequences homologous to the pTRACA22 RelE (ORF22-6, **Figure 1, Table S1**) derived from combined human gut metagenomes, plasmids and bacterial genome sequences, were used for construction of a phylogenetic trees. Symbols preceding sequence names denote the origin of the RelE sequence as detailed in the figure legend. Major clades predominantly populated with sequences of common phylogenetic origin are indicated by brackets: **A**) *Firmicutes ***B**) *Bacteroidetes/Chlorobi*, **C**) Plasmid **D**) *Proteobacteria ***E**) *Actinobacteria*. Numerals indicate nodes with bootstrap values of 40 or over (based on 1000 replicates). Scale bar indicates 0.1 amino acid substitutions per site. Accession numbers for chromosomal and plasmid sequences represented in the phylogenetic tree, along with those for gut metagenomic sequences encoding each RelE "type" are listed in **Table S2**.

Construction of phylogenetic trees using the aligned RelE sequences showed that the 33 distinct metagenomic RelE "types" are associated with all major bacterial divisions in the human gut (*Firmicutes*, *Actinobacteria *and *Bacteroidetes*, Fig 6). The pTRACA22 RelE was most closely related to sequences from *Blautia hydrogenotrophica *DSM 10507 (RUMHYD_01668), but was also closely related to sequences from human gut metagenomes (Type #1 and #2) and the *E. coli *pARS3 plasmid (Fig 6, Table S2). The association of the *E. coli *pARS3 RelE with pTRACA22 and other gut derived sequences in our phylogenetic analysis (Fig 6), rather than sequences originating in *E. coli *(the natural host of pARS3), other Gram negative enteric species, or other plasmid encoded sequences, indicates that this plasmid may be closely related to pTRACA22.

Furthermore, both chromosomally encoded and plasmid encoded sequences originating in Gram negative enteric genus such as *Escherichia*, *Salmonella*, *Shigella*, and *Yersinia *appeared distinct from both human gut metagenomic sequences, and those derived from draft genomes of Gram negative and Gram positive gut associated species (Fig 6). In particular, plasmid encoded RelE were observed to form a discrete clade, and were largely distinct from the chromosomally encoded RelE represented in our dataset (Fig 6). Similar observations have been noted previously for the CcdAB TA system, where plasmid encoded and chromosomally encoded toxins formed distinct clusters in phylogenetic analyses [35]. While it is unclear whether the sequences obtained from human gut metagenomes are encoded chromosomally or by plasmids, as chromosomal DNA represents the vast majority of the metagenomic datasets utilised it is most likely that chromosomally encoded TA modules have been retrieved.

### The *E. coli *pARS3 IS26 composite transposon is closely related to pTRACA22

**Figure 7:**
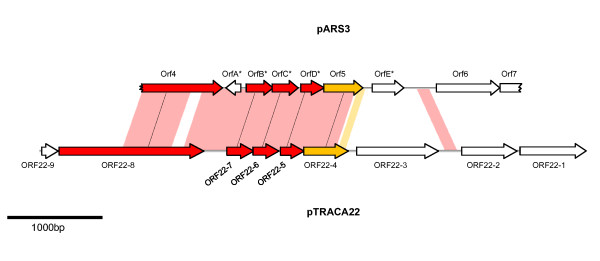
**Annotation of the available pARS3 IS26Tn sequence fragment and comparison with pTRACA22**. The sequence fragment of the *E. coli *plasmid pARS3 described by Wachino *et al*[34] was downloaded from GenBank [AB261016]. Due to the high identity of pTRACA22 ORFs with regions of the pARS3 sequence observed during tBlastn searches, the currently available pARS3 sequence (limited to a 3964 bp region of the 9.1 Kb IS26Tn) was analysed using Glimmer v3, and predicted ORFs annotated. All ORFs described by Wachino *et al *[34] were predicted along with five ORFs not originally described or annotated in the GenBank record, * denotes ORFs in pARS3 annotated during this study. Nucleotide sequences of pTRACA22 and pARS3 were compared using the Artemis Comparison Tool (ACT) and shaded areas between sequence maps represent regions of high identity at the nucleotide level. Areas shaded pink range from 95-97% identity, and areas shaded orange exhibited 89% identity. ORFs described previously by Wachino *et al *follow nomenclature used in that study [43], and the following functions were originally assigned to these ORFs: *Orf*4 - putative replication protein, *Orf*5: hypothetical protein, *Orf*6 - NpmA aminoglycoside resistance gene, *Orf*7 - putative ABC transporter substrate binding protein. ORFs are shown as arrows. Open arrows denote ORFs that do not have a homologue in both sequences. Coloured arrows indicate ORFs with homologues present in both pTRACA22 and pARS3, and "paired" ORFs are connected by dashed lines with colours representing % identity at the amino acid level: Red arrows indicate greater than 90% identity between paired ORFs, Orange arrows indicate greater than 80% identity between paired ORFs. Jagged edges indicate truncated ORFs.

Due to the close association between the pTRACA22 and pARS3 RelE observed in our phylogenetic analysis, the potential relationship between these plasmids was further explored. Comparison of pTRACA22 and the available pARS3 sequence (limited to a 3964 bp region of the plasmid; [Genbank: AB261016]) using the Artemis Comparison Tool (ACT) revealed that pTRACA22 shares high identity with a region of pARS3 encoding a 9.1 Kb IS26 based composite transposon (IS26Tn). Notably the regions of pTRACA22 encoding the plasmid replication protein (ORF22-8) and the RelBE TA addiction module (ORF22-6, and ORF22-7) exhibited very high identity to the available pARS3 IS26Tn region at the nucleotide level, but no homology to other plasmids from *E. coli *or closely related enteric organisms was observed for the IS26Tn sequence (Fig 7).

As several of the pTRACA22 ORFs mapped to the homologous region of pARS3 did not correspond to any ORFs identified during the original characterisation of this plasmid [34], or currently annotated in the Genbank record, the available pARS3 sequence was re-annotated for this study (Fig 7). This analysis identified several additional ORFs with high identity to ORFs encoded by pTRACA22, and revealed similar gene architecture (Fig 7). Wachino *et al*. (2007) [34] also reported the presence of a mobilization protein in the pARS3 IS26Tn designated *orf8*, which was truncated by the insertion of an IS26 element. It is likely that this corresponds to the pTRACA22 mobilisation protein ORF22-2, but as the sequence data for these regions of the pARS3 IS26Tn are not currently available, this hypothesis cannot be confirmed.

In contrast to the pARS3 IS26Tn, pTRACA22 does not encode the *npmA *aminoglycoside resistance gene or the adjacent ABC transporter binding protein (*orf6*, and *orf7 *in pARS3 respectively, as described by Wachino *et al*. [34]) and the region of the pARS3 IS26Tn encoding these shows no homology to pTRACA22. Furthermore, no IS26 elements were detected in pTRACA22.

Overall the pARS3 IS26Tn appears to have originated from a plasmid related to pTRACA22, and encodes genes with high identity to those found in the draft genomes of gut associated *Firmicutes*, particularly *B. hydrogenotrophica *DSM 10507 (Fig 4, Fig 6). This also raises the possibility that the novel aminoglycoside resistance mechanism encoded by the pARS3 IS26Tn originated in the human gut microbiome. However, tBlastn searches of the human gut datasets used for comparative metagenomic analysis [9,10] did not yield any significant hits to the pARS3 *npm *A protein.

## Discussion

In this study we have applied a metagenomic approach to isolate and characterise novel plasmids from the human gut microbiome. These plasmids ranged between 3.7 and 10.8 kb, and at present it is unclear if this size range represents a limitation of the TRACA system used to isolate them, a pre-dominance of smaller plasmids in the gut microbiome or a combination of these factors [12]. The range of G+C contents of plasmids isolated corresponds with the overall range in genomic G+C content observed among the major bacterial divisions comprising the human gut microbiota. The *Actinobacteria *constitute a major phylogenetic division within the human gut comprising high G+C Gram positive species [2,6]. In contrast other major phylogenetic groups, *Firmicutes *and *Bacteriodetes*, are comprised of low G+C Gram-positives and Gram-negatives respectively [2,6]. As such plasmids of a high G+C content (pTRACA10, pTRACA18) are likely to originate from bacteria belonging to the *Actinobacteria*.

Of the six complete plasmid sequences utilised in this study, no significant homology to pTRACA17, pTRACA20 or pTRACA30 was identified in any of the metagenome data sets examined. This may simply be due to a low abundance of these plasmids in the human gut microbiota, the generally low coverage of bacterial communities offered by current metagenomic data sets, or may reflect intra-individual variation in plasmids associated with the gut community. Alternatively, the apparent absence of these or closely related plasmids in the human gut metagenomes examined, may be attributed to a general incompatibility of plasmid DNA with the standard metagenomic approach used to generate many of the currently available datasets, which is focused on acquisition and sequencing of bacterial genomic DNA [7,9-13,33,36,37].

In contrast sequences homologous to plasmids pTRACA10 and pTRACA22 were identified in multiple human gut metagenomes. In particular, pTRACA22 appeared well represented in the combined human gut metagenomes analysed, and the absence of nucleotide sequences homologous to this plasmid in the non-human datasets, coupled with its presence in human gut datasets from geographically isolated individuals with a broad global distribution, suggests that pTRACA22 may be unique to the human gut microbiota. This has also been observed for bacteriophage present in the human gut community and recently Ebdon *et al*. (2007) reported that bacteriophages isolated from human faecal material were specific to the human gut, and absent in the general environment, as well as faecal samples from horses, sheep, pigs, cattle, rabbits and poultry [38]. Furthermore the general enrichment of certain MGE in the human gut microbiome has also been reported in recent metagenomic studies [10].

Investigation of the relative abundance of functions encoded by pTRACA10 and pTRACA22 revealed that several show a higher abundance in the human gut microbiome (Fig 3a). In particular the increased prevalence of the pTRACA22 TA addiction module in the human gut microbiome was unexpected, and the significance of this observation is currently unclear. TA modules have been shown to contribute to plasmid stability and maintenance through a post segregational killing mechanism, and consist of a stable toxin component which is inactivated by an unstable antitoxin [29,32,39]. Loss of the plasmid from daughter cells ultimately results in a loss of the antitoxin component and the killing of plasmid free cells [29,32,39]. In the case of the RelBE system, RelE encodes an RNA interferase which cleaves mRNA in a site specific manner at ribosomes, blocking translation [40,41]. Plasmid encoded TA modules are also involved in plasmid-plasmid competition [42], and plasmids harbouring a TA module have been shown to outcompete counterparts from the same incompatibility group lacking a TA module [42]. These attributes of TA modules may facilitate the maintenance of plasmids, or other DNA molecules harbouring them, in the human gut microbiome, and contribute to the observed prevalence of RelBE modules in this ecosystem (Fig 3). However, this explanation does not account for the lower prevalence observed for non-human metagenomes.

TA modules that are active and functional in surrogate cloning hosts may also generate bias in metagenomic libraries by enhancing stabilisation of constructs (such as metagenomic clones) harbouring them. Any such effects of TA modules could also account for the increased prevalence of the pTRACA22 RelBE and homologous modules in the human gut microbiome, as well as the differences between the relative abundance of toxin and antitoxin homologs (Fig 3). However, considering that orphan RelE toxin genes were identified on sequences retrieved from human gut metagenomes, and no increase in relative abundance was observed for the other TA modules analysed in this study, this explanation seems unlikely (Table S2).

It is also possible that bias between the metagenomic data sets due to variation in methods for construction of metagenomic libraries, such as efficiency of DNA extraction protocols, may account for the observed differences in prevalence of plasmid encoded functions between human and non-human metagenomes. However, the data sets utilised in this study were generated using comparable approaches, and in the case of the 15 human gut metagenomes utilised, which were generated by 2 distinct research teams [9,10], no demarcation between data sets from different groups was identified during our analysis. This is despite the observed paucity of sequences from *Bacteriodes *sp. in the metagenomes generated by Gill *et al*. (2006) [9]. Furthermore, analysis of other TA modules revealed a similar prevalence among the all metagenomic data sets, with few significant differences between human and non-human data sets (Fig 3c).

Plasmids and other MGE are also potentially involved in the maintenance of ecosystem functions, and retention of genes within this community in the absence of any selective pressure for the functions they encode. Many plasmids are highly stable even when providing no obvious survival advantage to the host cell, and numerous explanations have been suggested for the apparent stability of plasmids encoding traits such as antibiotic or heavy metal resistance, in the absence of direct selective pressure. These include the existence of unidentified selective pressures acting directly on the resistance or other genes encoded by a plasmid, or provision of a survival advantage sufficient to overcome the metabolic burden imposed on plasmid carrying cells, when selective pressure is removed [43,44].

However, TA modules may also constitute a mechanism by which plasmids and the genes they encode are retained in the absence of direct selective pressure [12]. It has also been proposed that chromosomally encoded TA modules may stabilise regions of adjacent DNA and maintain their vertical transmission [32,45,46]. Thus both plasmid and chromosomally encoded TA modules may be important in maintaining functional stability of microbial ecosystems such as the human gut microbiome.

The wide distribution and high prevalence of chromosomally encoded TA modules in both bacteria and archaea (particularly in free living species) has also lead to the hypothesis that these systems are advantageous to the host bacterium [32,47]. Since the majority of sequence data in the metagenomic libraries utilised will be derived from chromosomal DNA, most of the RelBE TA modules identified in the human gut microbiomes will be chromosomally encoded. Therefore it is also plausible that the increased abundance of these TA modules in the human gut microbiome is due to effects on the fitness of the bacterial host. Interestingly, while assessment of other TA modules did not reveal the prevalence observed for the pTRACA22 RelBE, the MazF toxin, which also functions as a ribonuclease and blocks translation by the same mechanism as RelE [32,41,48], did exhibit an increased prevalence in the human gut microbiome as well as other environments, albeit to a lesser extent (Fig 3c). Diverse functions have been proposed for chromosomally encoded TA modules in bacteria and archaea, and it is possible that these functions are also important in gut associated bacterial species.

RelBE and MazEF TA modules have been shown to modulate gene expression, undertake quality control of this process, and facilitate bacterial survival under nutrient limiting conditions or other environmental stresses [32,48,49]. The involvement of TA modules in gene expression may also be relevant to adaptation of prokaryotes to new environments, and TA modules have been implicated in the formation of persister cells which can survive exposure to antimicrobials and other stresses that are otherwise fatal [50]. It has also been proposed that chromosomally encoded RelE and ParE toxins are exploited as cellular regulators, and that the acquisition of these elements through HGT mediates the rapid development of novel regulatory pathways which facilitates adaptation to new environments through modulation of gene expression [47]. The formation of persister cells and the development of new regulatory pathways via TA have important implications for colonization of the gut, as well as survival of bacterial cells in the external environment, or during transmission to new hosts. However, the exact function of RelBE TA modules in bacteria inhabiting the human intestinal tract is currently unknown, and will require further detailed study to elucidate.

Interestingly, no RelE sequences homologous to the pTRACA22 RelE were identified in gut associated archaeal species. Since TA modules such as RelBE have been observed as highly prevalent and widely distributed in free living archaea, and were identified in bacterial species from all major phylogenetic divisions in the gut microbial community, the lack of sequences homologus to the pTRACA22 RelE in dominant gut archaeal species (*M. smithii *and *Ms. stadmanae*) was surprising. This may reflect differences in selective pressures imposed on bacteria and archaea colonising the human gut, or the adoption of alternate strategies to cope with the same environmental stress. While the exact nature and source of the observed difference between gut archaea and bacteria remain unclear, the identification of functions shared or distinct to each lineage will be important in developing a full understanding of the gut microbiota, and the contribution of constituent species to the development and output of this community.

The putative RelBE TA addiction module identified on pTRACA22 also has potential to impact on eukaryotic cells present in the human gut through activity of the RelE toxin component. While no eukaryotic homologues of RelE have been identified, this toxin has been shown to be active against both prokaryotic and eukaryotic organisms. The RelE toxin is functional in eukaryotic cells and has been shown to induce apoptosis in cultured human cell lines [51], inhibit the growth of yeast [52], and to cleave eukaryotic mRNA in the A site of eukaryotic ribosomes in a similar fashion to that described in bacteria [53]. However, studies demonstrating the activity of RelE in eukaryotic cells are currently limited to artificial expression of the toxin using a specialised vector [51,52]. As such further study is required to identify the *in vivo *effect, if any, of RelE toxins on eukaryotic cells, particularly in light of the increased prevalence of this TA module in the gut human gut microbiome.

As well as a wide distribution among human metagenomic data sets, pTRACA22 was also found to illuminate the transfer of genetic material between diverse members of the gut microbiota (Fig 7). The high identity of pTRACA22 to the *E. coli *pARS3 IS26-like transposon, indicates that this region of pARS3 is derived from a plasmid closely related to pTRACA22 (Fig 7), raising several intriguing possibilities. In particular the presence of the pTRACA22 putative replication protein on the pARS3 IS26Tn may allow pARS3 to function as a natural shuttle vector and replicate in disparate bacterial hosts. Considering the high identity of pTRACA22 encoded genes to *B. hydrogenotrophica*, this scenario would likely encompass replication in both Gram -ve and Gram +ve species. Furthermore, the presence of a RelBE addiction module (with high identity to that encoded by pTRACA22) in the pARS3 IS26Tn could stabilise pARS3 during transition between bacterial hosts, and provide this plasmid with a competitive advantage over other related elements in the same incompatibility group [29,35,39,42].

While the site of gene exchange that resulted in the formation of the pARS3 IS26Tn cannot be determined with complete certainty, several factors indicate the human or mammalian GI tract: i) Both species involved in the genetic exchange (*E. coli *and a gut associated *Firmicute *sp.) naturally inhabit the human gut; ii) The high oxygen sensitivity of many cultivated *Firmicutes *comprising the gut microbiota points to an anaerobic environment such as the human gut [54]; iii) The paucity of sequences homologous to pTRACA22 in the non-human gut metagenomes analysed here. Overall the high identity of pTRACA22 with the pARS3 IS26Tn supports the hypothesis that the human gut is a potential hotspot for HGT between disparate bacterial species [12,20,22,55], and highlights the potential for gene transfer from commensal organisms to clinically relevant pathogenic speices.

In light of the observed prevalence of RelBE TA modules in the human gut microbiome, the reported functionality of the RelE toxins in eukaryotic cells, and the potential role of this system in mediating bacterial survival in the gut environment, the RelBE module encoded by pTRACA22 merits further investigation, and these studies are currently underway in our laboratory. Furthermore, continued analysis of the mobile metagenomes associated with microbial communities will be important in understanding many aspects of microbial community function and evolution. A greater knowledge of the MGE associated with bacterial communities will also facilitate the development of novel strategies and tools to dissect and manipulate these complex ecosystems.

## Conclusions

In this study we demonstrate the application of a metagenomic approach to isolate and analyse plasmids resident in the human gut microbiota. The use of the culture independent TRACA system to capture novel plasmids from the human gut mobile metagenome, coupled with subsequent comparative metagenomic analysis of complete plasmid nucleotide sequences have highlighted the increased prevalence of unexpected functions in the gut ecosystem, and provided new insights into plasmids associated with the human gut microbiome.

The existence of plasmids encoding functions prevalent in the gut microbiome demonstrates the mobility of the relevant genes within this community, and considering the proposed role of HGT in the convergence and expansion of enriched community gene sets [7,56], the identification and characterisation of such plasmids will be important in understanding the development and evolution of the human gut microbiome. Furthermore, as plasmids and other MGE frequently encode genes advantageous to survival in a particular environment, the analysis of plasmid encoded genes from the human gut mobile metagenome is likely to provide important insights into challenges faced by microbes colonising the human gut, and the mechanisms by which these are overcome.

## Methods

### Preparation of metagenomic DNA

Plasmids were acquired from the gut microbiome of a healthy 26 year old British male Caucasian volunteer, on a typical western style diet. The subject had not recently consumed probiotics, and had not received antibiotic treatment for over 3 years prior to collection of faecal samples. The protocol was approved by the Clinical Research Ethics Committee of the Cork Teaching Hospitals, and written informed consent was obtained from the subject for participation on the study and publication of data generated. Bacteria were harvested using a Nycodenz (Axis-Shield, Italy) density centrifugation gradient and subsequently total metagenomic DNA was extracted using the Genomix Cells & Tissues extraction kit (Talent, Italy) according to manufacturer's instructions. Approximately 1 μg of metagenomic DNA was treated with ATP-dependant Plasmid Safe DNase (Epicentre, USA) according to manufacturer's instructions, to remove sheared genomic DNA. Plasmid Safe digests were incubated overnight at 37°C and subsequently inactivated by heating at 70°C for 30 min followed by chilling on ice, and were used in plasmid capture reactions without further processing.

### Isolation of novel plasmids from the human gut mobile metagenome

Plasmids were isolated from plasmid-safe treated metagenomic preparations using the culture independent TRACA system as previously described [13]. TRACA reactions were set up using the EZ-*Tn5 *OriV Kan2 element (Epicentre, USA). Each reaction was composed of 35 μl plasmid-safe digested metagenomic DNA, 2 units of transposase, 1 × EZ-*Tn5 *reaction buffer and brought to a final volume of 50 μl by addition of sterile deionised water. Reactions were incubated for 2 h at 37°C and stopped by addition of 5 μl EZ-*Tn5 *stop buffer and heating at 70°C for 15 min. Reactions were subsequently diluted by addition of 450 μl sterile deionised water and purified using 100 kDa molecular weight cut off columns (Millipore, USA) according to manufacturer's instructions. Reactions were ultimately reduced to 10 μl, and 5 μl of this was used to transform *E. coli *EPI300 competent cells (Epicentre, USA) by electroporation (18 kV/cm, 200 Ω resistance, 25 μF capacitance). Transformants were selected using LB agar supplemented with 50 μg/ml kanamycin.

### Plasmid sequencing, annotation and sequence analysis

Plasmids pTRACA 20, 22 and 30 were sequenced by GATC biotech AG (Germany) using a primer walking strategy, while pTRACA18 was sequenced by the Wellcome Trust Sanger Centre (Cambridge, UK). Open reading frames encoded by these plasmids were predicted using Glimmer 3 [57,58]. Their putative functions assigned based on homologies to proteins and conserved domains identified in BlastP, tBlastn, and rpsBlast searches. For BlatsP and tBlastn searches the nr dataset was utilised (which encompasses the following public databases: All non-redundant GenBank, RefSeq proteins, PDB, SwissProt, PIR, PRF) and all sequences assigned a putative function generated hits with an e-value of 1e^-02 ^or lower. For rps-Blast searches the NCBI Conserved Domains Database (CDD) was searched (which is composed of NCBI annotated entries plus protein domain models from Pfam, SMART, COG, PRK, TIGRFAM databases) and hits used to assign putative functions generated e-values of 1e-^04 ^or lower. Plasmid sequences were annotated using Artemis [59] and genetic maps constructed using Vector NTI software [60]. The annotated sequences have been deposited in Genbank under the following accession numbers: FN429765 - FN429568 (pTRACA18 to pTRACA30 respectively). Assessment of similarity between pTRACA22 and the available pARS3 plasmid sequence was undertaken using the web version of the Artemis Comparison Tool (WebACT), with the user defined sequences facility [61].

### Comparative metagenomic analysis

Our comparative metagenomic analysis utilised 15 human gut metagenomes [9,10], the combined gut metagenomes of lean and obese mice [11], as well as soil and marine metagenomes [30,31]. Using these datasets three complimentary surveys were undertaken to investigate various aspects of plasmid distribution and prevalence of encoded functions: i) Detection of nucleotide sequences homologous to plasmids utilised in this study; ii) Investigation of the relative abundance of functions encoded by pTRACA10 and pTRACA22 between human and non-human metagenomes; iii) Investigation of the variation between individual human gut metagenomes, with respect to functions encoded by pTRACA10 and pTRACA22.

i) Initially the combined human gut metagenomes, and all non-human metagenomes were searched using Blastn to identify nucleotide sequences homologous to plasmids analysed in this study (pTRACA10, 17, 18, 20, 22 and 30). Only sequences 100 bp or greater in length with an identity of 80% or over and e-value of 1e^-5 ^were considered significant in these searches (Fig 2, Table 1).

ii) The relative abundance of plasmid encoded functions, and homologues of various TA modules, in human and non-human metagenomic datasets was examined as previously described [7]. Metagenomes were searched using tBlastn to identify amino acid sequences homologous to plasmid encoded ORFs or TA module genes (Fig 3a and 3c). Hits generating an e value of 1e^-5 ^or lower and with a length of 30aa or over were used to calculate the hits/Mb for subject sequences in each search (i.e. each plasmid encoded ORF or toxin/antitoxin amino acid sequence) [7]. For assessment of MazEF, ParDE and HigBA TA modules, amino acid sequences originating from *Escherichia coli *O157_H7_EDL933 [GenBank GI: 16445223] were used to search metagenomic datasets, as this species encoded all three modules and *Proteobacteria *are prevalent in all three environments examined (mammalian gut, soil, marine). The significance of the observed differences between human and non-human metagenomes was explored using the χ2 distribution, and the approximate significance at the 99% confidence interval determined using Microsoft Excel.

iii) To assess the general distribution and variation in plasmid encoded functions between individual human gut metagenomes, the top hits (based on bitscore) to each plasmid ORF from tBlastn searches of each individual gut metagenome were retrieved, and the incidence as well as % identity to the relevant plasmid encoded ORF was recorded (Fig 3b). Only hits generating an e-value of 1e^-5 ^or lower and with a length of 30aa or over were utilised.

### RelE sequence acquisition and construction of phylogenetic trees

Toxin components of RelBE modules were selected for this analysis as orphan toxin genes have been reported in a wide variety of bacteria [32]. To retrieve complete RelE amino acid sequences from combined gut metagenomes, nucleotide sequences identified as encoding *relE *genes were acquired from human gut metagenomes. ORFs homologous to both *relE *and *relB *genes from pTRACA22 were identified using tBlastn, and complete ORFS annotated using Artemis. For the construction of phylogenetic trees, sequences which contained errors or inappropriate stop codons were removed from the data set, along with sequences that lacked intact COG3041 conserved domains observed in the pTRACA22 and other RelE sequences. In addition, potentially truncated ORFs were also removed and identical sequences resolved to a single representative (Table S2). The resulting 33 distinct RelE sequences were combined with sequences homologous to the pTRACA22 putative RelE retrieved using BlastP and tBlastn searches of the public databases, and aligned using Clustal_W. Subsequently unrooted phylogenetic trees were constructed with the neighbour-joining algorithm and p-distances model (1000 bootstrap replicates), using the MEGA3 software package [62].

## List of Abbreviations

MGE: Mobile genetic elements; TRACA: Transposon aided capture; BLAST: Basic local alignment search tool; TA module: Toxin/antitoxin addiction module; HGT: horizontal gene transfer; ORF: Open reading frame.

## Authors' contributions

BVJ conceived the project. BVJ and JRM coordinated the project. BVJ, FS performed all experimental work and analysed the data. BVJ wrote the manuscript. BVJ and JRM edited the manuscript. All authors have read and approved the manuscript.

## Supplementary Material

Additional file 1 **Table S1 - Detailed summary of pTRACA18, pTRACA20, pTRACA22, and pTRACA30 open reading frames and putative functions**. Detailed information on putative functions and homologies of predicted open reading frames identified in plasmids pTRACA18, pTRACA20, pTRACA22 and pTRACA30. Click here for file

Additional file 2 **Table S1 - Sequences represented in Figure 6**. Accession numbers of RelE amino acid sequences used in Figure 6. Sequences retrieved from human gut metagenomic datasets are included and those harbouring complete putative RelBE modules, orphan RelE toxin genes, and potentially novel antitoxin components are indicated. Click here for file
