# Supplementary material for: Comparative metagenomic analysis of plasmid encoded functions in the human gut microbiome
Source: BMC Genomics. 2010 Jan 19;11:46. doi: 10.1186/1471-2164-11-46 (PMC2822762; doi:10.1186/1471-2164-11-46)
Supplement: Additional file 1 — Table S1 - Detailed summary of pTRACA18, pTRACA20, pTRACA22, and pTRACA30 open reading frames and putative functions. Detailed information on putative functions and homologies of predicted open reading frames identified in plasmids pTRACA18, pTRACA20, pTRACA22 and pTRACA30. [file 1471-2164-11-46-S1.DOCX]

**Table S1 - Summary of pTRACA18, pTRACA20, pTRACA22, and pTRACA30 open reading frames and putative functions.**

| **Plasmid**  **(Source)** | **ORF** | **Location^1^** | **Length of amino acid sequence^2^** | **Putative product/function^3^** | **Alignment**  **and identity^4^** |
| --- | --- | --- | --- | --- | --- |
| **This study** | | | | | |
|  | | | | | |
| **pTRACA18** |  |  |  |  |  |
|  | ORF18-1 | 4-201 | 65aa | Unknown function. | NSH |
|  | ORF18-2 | 272-544 c | 90aa | Unknown function. Similar to hypothetical protein HMPREF0577_1032 from *Mobiluncus mulieris* ATCC 35243. NCD. | 57% (49/85) |
|  | ORF18-3 | 550-810 c | 86aa | Unknown function. Similar to hypothetical protein COLINT_03765 *Collinsella intestinalis* DSM 13280. NCD. | 33% (29/86) |
|  | ORF18-4 | 1165-2571 | 468aa | Putative replication protein. Similar to EUBIFOR_02271 *Eubacterium biforme* DSM 3989 and putative replication protein from *Staphylococcus sciuri* plasmid PSCFS1. Conserved domains of HTH_Hin_like (Helix-turn-helix domain of Hin, cd00569,) DNA-binding proteins detected*. | 45% (203/444) |
|  | ORF18-5 | 2748-3083 | 111aa | Unknown function. | NSH |
|  | ORF18-6 | 3050-3418 | 122aa | Unknown function. | NSH |
|  | ORF18-7 | 3530-3832 c | 100aa | Unknown function. | NSH |
|  | ORF18-8 | 4090-4302 c | 70aa | Unknown function. | NSH |
|  | ORF18-9 | 4279-5796 | 505aa | Putative MobA_MobL type plasmid mobilization protein. Similar to *Anaerotruncus colihominis* DSM 17241 predicted MobA/MobL protein (ANACOL_03249). Conserved domains detected from MobA_MobL superfamily (pfam03389), and the TraA_Ti, Ti-type conjugative transfer relaxase TraA (TIGR02768)**^M^**. | 37% (94/251) |
| **pTRACA20** |  |  |  |  |  |
|  | ORF20-1 | 102-1223 c | 373aa | Putative plasmid mobilization/recombination protein. Similar to a mobilization protein from *Geobacillus stearothermophilus*. Mob_Pre superfamily (pfam01076) conserved domains detected. | 42% (96/2228) |
|  | ORF20-2 | 1633-1749 c | 38aa | Unknown function. | NSH |
|  | ORF20-3 | 1712-2005 c | 97aa | Unknown function. | NSH |
|  | ORF20-4 | 2034-2216 c | 60aa | Unknown function. | NSH |
|  | ORF20-5 | 2275-3657 c | 460aa | Putative RepA type replication protein similar to RepA from *Campylobacter jejuni* pTet37, pCC31, and pTet. DNA_Primase_S (pfam01896), eukaryotic and archaeal DNA primase small subunit superfamily conserved domains detected. | 69% (312/448) |
| **pTRACA22** |  |  |  |  |  |
|  | ORF22-1 | 180-800 c | 206aa | Putative plasmid recombination protein. Similar to RUMHYD_01660 putative plasmid recombination protein from *Blautia hydrogenotrophica* DSM 10507. NCD. | 99% (186/187) |
|  | ORF22-2 | 832 - 1416 c | 194aa | Putative plasmid mobilization/recombination protein. Similar to predicted Mob_Pre type protein RUMHYD_01660 from *Blautia hydrogenotrophica* DSM 10507. Mob_Pre superfamily (pfam01076) conserved domains detected. | 98% (170/172) |
|  | ORF22-3 | 1659-2555 c | 298aa | Unknown function. Putative toxin protein. Similar to putative entericidin protein RUMHYD_01665 from *Blautia hydrogenotrophica* DSM 10507. NCD. | 100% (248/248) |
|  | ORF22-4 | 2610-3077 c | 155aa | Unknown function. Identical to hypothetical protein RUMHYD_01666 from *Blautia hydrogenotrophica* DSM 10507. NCD. | 100% (155/155) |
|  | ORF22-5 | 3074-3322 c | 82aa | Unknown function. Putative thiamine-phosphate pyrophosphorylase, similar to RUMHYD_01667 *Blautia hydrogenotrophica* DSM 10507. NCD. | 100% (82/82) |
|  | ORF22-6 | 3341-3613 c | 90aa | Putative addiction module toxin component. Similar to RUMHYD_01668 from *Blautia hydrogenotrophica* DSM 10507. Conserved domains of COG3041, TIGR02385, RelE_StbE, and pfam_05016 plasmid stability protein superfamilies detected. | 100% (90/90) |
|  | ORF22-7 | 3606-3887 c | 93aa | Putative addiction module anti-toxin component. Similar to RUMHYD_01669 from *Blautia hydrogenotrophica* DSM 10507. Conserved domains from RelB superfamily detected including RelB_DinJ antitoxin family (TIGR02384), RelB (pfam04221), and DNA damage inducible protein DinJ (COG3077). | 100% (93/93) |
|  | ORF22-8 | 4132-5658 c | 508aa | Putative plasmid replication protein. Similar to putative ATP-dependent DNA ligase RUMHYD_01670 from *Blautia hydrogenotrophica* DSM 10507, putative replication protein SmonDRAFT_14340 from S*treptobacillus moniliformis* DSM 12112and Rep protein from *Treponema denticola* plasmid pTS1_p1. NCD. | 99% (507/508) |
|  | ORF22-9 | 5662-5841 c | 59aa | Unknown function. Similar to hypothetical protein RUMHYD_01671 from *Blautia hydrogenotrophica* DSM 10507. NCD. | 99% (56/57) |
| **pTRACA30** |  |  |  |  |  |
|  | ORF30-1 | 2-568 c | 188aa | Putative ATPase involved in plasmid partitioning. Similar to ParA plasmid segregation protein from *Lawsonia intracellularis* plasmid 2. Conserved domains detected from ParA chromosome segregation proteins (cd02042)*****, and partial domains from ArsA ATPase efflux proteins (cd02035)*****, and Soj ATPases**^M^** (COG1192). | 58% (101/174) |
|  | ORF30-2 | 565-711 c | 48aa | Unknown function. | NSH |
|  | ORF30-3 | 708-941 c | 77aa | Unknown function. Similar to COPEUT_02663 hypothetical protein from *Coprococcus eutactus* ATCC 27759. Conserved domains from COG4443 and DUF2128 superfamily detected, comprising uncharacterized proteins conserved in bacteria. | 53% (36/67) |
|  | ORF30-4 | 922-1065 c | 47aa | Unknown function. | NSH |
|  | ORF30-5 | 1058-1996 c | 312aa | Putative tyrosine site specific integrase. Similar to *Streptococcus thermophilus* plasmid SMQ308 integrase. Conserved domains detected from XerC and XerD tyrosine site specific recombinase (PRK00236)**^M^** and the DNA_BRE_C superfamily of DNA breaking-rejoining enzymes. | 53% (169/314) |
|  | ORF30-6 | 2256-2885 c | 209aa | Hypothetical protein, similar to hypothetical protein CLOSTMETH_01823 from *Clostridium methylpentosum* DSM 5476. NCD. | 38% (67/172) |
|  | ORF30-7 | 2897-4862 c | 654aa | Putative DNA primase/helicase. Similar to putative primase/helicase of *Streptococcus thermophilus* plasmid SMQ308. Conserved domains from TOPRIM primases (cd01029)*****, ABC_ATPase superfamily ATP binding cassette with A and B walker domains, and P-loop nucleoside triphosphate hydrolases (cl09099). | 50% (276/543) |
|  | ORF30-8 | 4934-5254 c | 106aa | Putative DNA primase/helicase. Similar to primase/helicase predicted to be encoded by *Streptococcus thermophilus* plasmids pSMQ-316 and pSMQ308. NCD. | 42% (39/92) |
|  | ORF30-9 | 5251-5478 c | 75aa | Unknown function. | NSH |
|  | ORF30-10 | 5755-5940 c | 61aa | Unknown function. | NSH |
|  | ORF30-11 | 6006-6254 c | 82aa | Unknown function. | NSH |
|  | ORF30-12 | 6251-7390 c | 379aa | Unknown function. | NSH |
|  | ORF30-13 | 7390-9234 c | 614aa | Unknown function. Similar to hypothetical protein from *Streptococcus themophilus* pSMQ308. NCD. | 24% (49/202) |
|  | ORF30-14 | 9289-9786 c | 165aa | Unknown function. Similar to hypothetical protein WS2053 from *Wolinella succinogenes* DSM 1740. NCD. | 31% (28/90) |
|  | 30-ORF15 | 9802-9921 c | 39aa | Unknown function. | NSH |
|  | 30-ORF16 | 10074-10307 c | 77aa | Putative excisionase. Similar to hypothetical protein from *Streptococcus thermophilus* plasmid pSMQ308, *Enterococcus faecium* TX1330, and *Acholeplasma laidlawii* PG-8A putative excisionase. Conserved domains form the HTH_MerR-SF superfamily of excisionase proteins detected (TIGR01764). | 51% (27/52) |
|  | 30-ORF17 | 10313-10609 c | 98aa | Hypothetical protein. | NSH |
|  | 30-ORF18 | 10615-10827 c | 70aa | Putative ATPase. Similar to ATPase from *Lawsonia intracellularis* plasmid 2. Conserved domains from Soj ATPases detected**^M^**. | 54% (33/61) |
| **Jones & Marchesi (2007) [13]** | | | | | |
|  |  |  |  |  |  |
| **pTRACA10** |  |  |  |  |  |
|  |  |  |  |  |  |
|  | ORF10-1 | 1089-2600 | 503aa | Putative plasmid mobilisation protein. Similar to MobB mobilization protein from *Bifidobacterium longum* plasmid pKJ36. MobA/MobL conserved domains detected. | 43% (50/144) |
|  | ORF10-2 | 2597-3403 c | 268aa | Hypothetical protein. Unknown function. | - |
|  |  |  |  |  |  |
|  | ORF10-3 | 3331-4044 c | 237aa | Putative phophoesterase/phosphohydrolase. COG4168 conserved domains detected. Similar to putative phosphoesterase from *Aurantimonas* spp. | 33% (59/177) |
|  |  |  |  |  |  |
|  | ORF10-4 | 4681-6579 | 632aa | Putative plasmid replication protein. Similar to replication protein form *Treponema denticola* plasmid pTS1. No conserved domains detected. | 35% (145/413) |
|  |  |  |  |  |  |
|  |  |  |  |  |  |
| **pTRACA17** |  |  |  |  |  |
|  | ORF17-1 | 36-713 c | 225aa | Putative plasmid mobilisation protein. Similar to Mob protein form *Bacillus thuringiensis* plasmid. Pre_Mob conserved domains detected | 46% (81/174) |
|  | ORF17-2 | 1632-2129 | 165aa | Putative plasmid replication protein. Similar to replication protein form *Bacillus subtilis* plasmid pIM13. Conserved domains from RepL firmicute plasmid replication proteins detected. | 35% (47/131) |
|  |  |  |  |  |  |

The putative product/function of plasmid encoded ORFs were predicted using BlastP and conserved domain searches. As well as plasmids newly characterised in this study (pTRACA18,20,22,30), plasmids characterised previously by Jones *&* Marchesi (2007) (pTRACA10 and pTRACA17;) are also represented [13].

**1)** Provides the location of each predicted ORF in bp, c indicates ORF encoded on complementary strand.

**2**) Length of the predicted amino acid sequence from annotated ORFs, given as number of amino acid residues. **3**) Summary of Blast searches and predicted product/function of ORF. For rpsBlast searches: * denotes a specific hit, and **^M^** denotes hits to computationally detected multi-domains. Non-specific hits are reported as hits to the overall protein superfamily. Specific hits provide a very high confidence level in the association of query sequence and detected conserved domain(s) and high confidence level in inferred protein function. Non-specific hits are statistically significant and general protein function can be inferred from the protein superfamily. For non-specific-hits and multidomain hits inferred function is less certain. For rpsBlast conserved domain searches all e-values were 1e^-04^ or lower. **NCD** - no conserved domains detected. **4**) % identity of plasmid ORF amino acid sequences to homologous sequences in public databases identified in BlastP searches (e values = 1e^-02^ or lower). **NSH** - No significant hits generated. For plasmids characterised previously (pTRACA10 and pTRACA17) predicted functions and results of Blast are as described in the original publication [13].
