# Supplementary material for: Comparative metagenomic analysis of plasmid encoded functions in the human gut microbiome
Source: BMC Genomics. 2010 Jan 19;11:46. doi: 10.1186/1471-2164-11-46 (PMC2822762; doi:10.1186/1471-2164-11-46)
Supplement: Additional file 2 — Table S1 - Sequences represented in Figure 6. Accession numbers of RelE amino acid sequences used in Figure 6. Sequences retrieved from human gut metagenomic datasets are included and those harbouring complete putative RelBE modules, orphan RelE toxin genes, and potentially novel antitoxin components are indicated. [file 1471-2164-11-46-S2.DOCX]

**Table S2 - Sequences represented in Figure 6**

**Metagenomic Sequences**

Human gut metagenome Type 1 (Hum 7, Hum 8, In-D; AQK01007353.1, AAQL01008281, BABD01025538)

Human gut metagenome Type 2 (F2-V; BAAX01003632.1)

Human gut metagenome Type 3 (F2-Y; BABA01001718.1)**

Human gut metagenome Type 4 (F2-X; BAAZ01000274.1)

Human gut metagenome Type 5 (In-D, In-E, In-M, F2-X, F2-W, Hum8; BABD01003896.1, BABF01004551.1, BABE01003002.1, BAAZ01003320.1, BAAY01001658.1, AAQL01002402.1, AAQL01003492.1, AAQL01011777.1)

Human gut metagenome Type 6 (In-E; BABE01002191.1)

Human gut metagenome Type 7 (F2-W; BAAY01012034.1)

Human gut metagenome Type 8 (F2-V; BAAX01002814.1)

Human gut metagenome Type 9 (In-M; BABF01007926.1)

Human gut metagenome Type 10 (F2-W, F2-X; BAAY01017093.1, BAAZ01004500.1)*

Human gut metagenome Type 11(F2-W; BAAY01008353.1) *

Human gut metagenome Type 12 (In-E, InB; BABE01000165.1, BABE01005272.1, BABC01000115.1)

Human gut metagenome Type 13 (F2-V; BAAX01002779)

Human gut metagenome Type 14 (F1-S; BAAU01015487.1) *

Human gut metagenome Type 15 (F1-S, F1-T; BAAU01021241.1, BAAV01001101.1)

Human gut metagenome Type 16 (In-D; BABD01002050.1)

Human gut metagenome Type 17 (In-A; BABB01001970.1)*

Human gut metagenome Type18 (F1-T; BAAV01011393.1) *

Human gut metagenome Type 19 (F2-V; BAAX01008032.1)

Human gut metagenome Type 20 (F2-W; BAAY01024089.1)

Human gut metagenome Type21 (F2-Y; BABA01003547.1)**

Human gut metagenome Type 22 (F2-X; BAAZ0102138238.1)**

Human gut metagenome Type 23 (F2-W; BABE01004361.1)

Human gut metagenome Type 24 (Hum 8; AAQL01011274.1)

Human gut metagenome Type 25 (Hum 7; AAQK01004293.1)

Human gut metagenome Type 26 (F1-S; BAAU01000861.1)

Human gut metagenome Type 27 (F1-S; BAAU01010805 .1)

Human gut metagenome Type 28 (In-D; BABD01000003.1)**

Human gut metagenome Type 29 (In-E; BABE01001016.1)

Human gut metagenome Type 30 (In-M; BABF01000868.1)

Human gut metagenome Type 31 (F2-V; BAAX01009308.1)

Human gut metagenome Type 32 (In-R; BABG0102940.1)

Human gut metagenome Type 33(F1-U; BAAW01002559.1)

* Sequences in which no RelB was identified;

**sequences in which a candidate RelB antitoxin gene was identified but lacked RelB conserved domains

**Chromosomal sequences**

*Candidatus Protochlamydia amoebophilia* UWE25; pa1923 (YP 008911.1) #1

*Candidatus Protochlamydia amoebophilia* UWE25 pa1912 (YP 008922.1) #2

*Desulfitobacterium hafniense* Y51; DSY2033 (YP 518266.1)

*Enterococcus faecalis* V583; EF0513 (NP_814294.1)

*Treponema denticola* ATCC 33405, TDE 0303 (NP_970917.1 ) #1

*Treponema denticola* ATCC 35405; TDE0505 (NP 971119.1) #2

*Fusobacterium nucleatum* subsp. *vincentii* ATCC 49256 FNV1999s (ZP_00143340.1)

*Streptococcus pneumoniae* TIGR4; SP_0276 (NP_344814.1)

*Desulfitobacterium hafniense* Y51; DSY2033 (YP 518266.1)

*Clostridium nexile* DSM 1781*,* CLONEX 01755 (EEA82359.1)

*Clostridium bolteae* ATCC BAA-613, CLOBOL 04273 (ZP_02086730.1) #1

*Clostridium bolteae ATCC BAA-613; CLOBOL 05794* (ZP_02088197.1*) #2*

*Clostridium scindens* ATCC 35704; CLOSCI 01688 (ZP_02431468.1

*Ruminococcus obeum* ATCC 29174; RUMOBE 02609 (ZP_01964879.1)

*Blautia hydrogenotrophica* DSM 10507; RUMHYD_01668 (ZP_03782231)

*Desulfitobacterium hafniense* DCB-2; Dhaf_2707s (ZP_01369432.1)

*Flavobacteria bacterium* BAL38; FBBAL38 08919 (ZP_01734455.1)

*Parabacteriodes merdae* ATCC 43184; PARMER 00551 (ZP_02030579.1)

*Parabacteriodes distasonis* ATCC 8503; BDI 2692 (YP_001304033.1)

*Escherichia coli* K12, substr DH10B, ECDH10B_0207 (ACB01396.1)

*Escherichia coli* HS, EcHS_A0253 (YP_001457026.1)

*Escherichia coli* ATCC 8739; EcolC_3395 (YP_001726341.1)

*Shigella sonnei* Ss046; SSO_0267 (YP_309286.1)

*Escherichia coli* O157:H7; ECs0252 (NP_308279)

*Helicobacter pylori* 26695; HP0892 (NP_207685.1)

*Pelodictyon phaeoclathratiforme* BU-1 Ppha_2504s (YP_002019302)

*Sphingomonas sp*. SKA58; SKA58_18640 (ZP_01303660.1)

*Xylella fastidiosa* Ann-1; XfasoDRAFT_4618 (ZP_00684447.1)

*Yersinia enterocolitica* subsp. *enterocolitica* 8081; YE1929 (YP_001006185.1)

*Salmonella enterica* subsp. *enterica* serovar Dublin SeD_A3886 (YP_002217476)

*Salmonella typhimurium* LT2, (NC_003197)

*Salmonella enterica* subsp. *enterica* serovar Saintpaul SeSPA_A4135 (ZP_03165884)

*Bifidobacterium longum* subsp. *infantis* ATCC 15697; Blon_1399 (YP_002322863.1)

*Bifidobacterium adolescentis* L2-32; BIFADO 00434 (ZP_02028024.1)

**Plasmid sequences**

pTRACA22 (FN429767)

*Escherichia coli* pARS3 (AB261016)

*Helicobacter pylori* plasmid pHel5 (AAM22665.1)

*Helicobacter pylori* CCUG 17874*,* plasmid pHP666 (ABA26015.1)

*Delftia acidovorans* plasmid pUO1 transposon TnHad2 (AB063332.1)

*Xylella fastidiosa* plasmid pXF5823 (AF322908.1)

*Sphingomonas wittichii* RW1 plasmid pSWIT01 (ABQ71228.1)

*Pseudomonas syringae* pv. *phaseolicola* 1448A small plasmid (CP000060.1)

*Achromobacter xylosoxidans* plasmid pA81 (AJ515144.2)

Uncultured bacterium IncP-1beta plasmid pB10 (AJ564903.1)

*Ralstonia eutropha* JMP134 plasmid 1 (CP000093.1)
